# Do inequalities exist in the disadvantaged populations? Levels and trends of full and on-time vaccination coverage in two Nairobi urban informal settlements

**DOI:** 10.1016/j.gloepi.2020.100044

**Published:** 2020-11

**Authors:** Martin K. Mutua, Shukri F. Mohamed, Samuel Iddi, Sylvia Muyingo, Bonventure Mwangi, Damazo Kadengye, for the Nairobi Urban Health Demographic Surveillance System

**Affiliations:** a African Population and Health Research Center, APHRC Campus, Manga Close, Off Kirawa Road, P.O. Box 10787-00100, Nairobi, Kenya; b Department of Statistics and Actuarial Science University of Ghana P. O. Box LG 115 Legon, Accra Ghana

**Keywords:** On-time immunization, Inequality, Fully immunization, Concentration index, Recurrence survival analysis

## Abstract

There has been an improvement in childhood vaccination coverage over the last two decades worldwide. However, inequities exist among different populations. Vaccination programs should focus not only on increasing coverage but as also in timeliness to ensure maximum protection. This study examined the levels, inequities, and trends of full and on-time vaccination coverage in two urban informal settlements in Nairobi. The study used longitudinal data from the Nairobi Urban Health and Demographic Surveillance System from 2003 to 2017 to estimate full and on-time vaccination coverage and assess inequalities by background characteristics. The frailty shared Cox model was used to assess time to full- and on-time- immunization coverage. Out of 32,018 children aged 12 to 59 months, less than half (46.7%) produced a vaccination card during the interview. Full and timely immunization coverage was higher in Viwandani site, among Kikuyu and Kamba ethnic groups, and children from the richest quintile. Timely vaccination was below 50% throughout the survey periods. After accounting for the intragroup correlations, for a given level of frailty, the hazard for being fully immunized was 10% more likely among the wealthiest compared to the poorest children. The hazard for being fully immunized was 16%, 16% to 19% less likely for Luhya, Luo, and others as compared to the Kikuyu ethnicity respectively. In conclusion, the study has shown that coverage has been increasing over the years but inequalities exist in immunization coverage among the most disadvantaged populations. More focused intervention approaches that target the disadvantaged groups are needed.

## Introduction

Immunization programs are considered the most cost-effective interventions in the world [28]. Child mortality and morbidity due to vaccine-preventable diseases (VPDs) has tremendously reduced in the last half a century [8,16,26,41] and can be attributed to the Expanded Program on Immunization (EPI). The success of the program is evident in developing countries where coverage has increased substantially over the past decade. However, child mortality and morbidity rates are still relatively high in sub-Saharan Africa (SSA) compared to other regions though there have been huge reductions in the burden of VPDs. Since the launch of the EPI program in 1974, most of the countries especially the developing countries have seen the introduction of vaccines against key VPDs; including tuberculosis, Diphtheria, pneumonia, pertussis, yellow fever, poliomyelitis, measles, tetanus and rotavirus, infections. All the routine vaccines are provided to every child at no cost in all developing countries.

The success of the program in terms of how many children get vaccinated has always been measured using coverage; the proportion of eligible children receiving a vaccine. Globally, coverage of the third dose of Diphtheria, Pertussis, and Tetanus (DPT3) has been used to measure the success of vaccination programs. Fully immunized child (FIC) - a child receiving all the recommended routine vaccines by the first year of life - has also been used in survey studies to measure program success.

Routine vaccination coverage (measured by the proportion of children under five years old receiving 3 doses of DPT containing vaccines by age of 12 months has been on the rise since the launch of EPI program across all regions in the world, from 5% in 1974 to 84% in 2013 and 85% in 2017 [6,42]. Even though national vaccine coverages from developing countries still lag, there has been an overall steady increase in the last decade. However, the steady increase has not been consistent among the different sub-populations especially the hard-to-reach populations; populations in rural areas, populations with low education level, populations in the lowest wealth quintile [33] and populations in the urban informal settlements [4,[11]. All efforts have been geared at addressing these inequality gaps by improving coverage and ultimately attaining the Global Vaccine Action Plans (GVAP) targets [45]. The WHO estimates that around 19.9 million children remain unvaccinated with the DPT3 vaccine of which the majority are from the developing countries. It is estimated that one out of 5 children or 20% of children in the African region do not receive the vaccines they need [46]. Studies have documented the levels of vaccine coverage as well as the inequality existing in terms of vaccine coverage [33].

Vaccination programs mainly focus on increasing coverage and reaching hard-to-reach children. Over the years, the coverage and reach of these programs have been successful. However, of equal importance, is the timeliness of vaccination (defined as children receiving their vaccines more than four days before - or four weeks after the target date) which has not received similar attention. Delays in vaccinating a child mean the child remains vulnerable to specific infections for a longer period and hence a higher chance of being susceptible to VPDs. To have a quality immunization program that provides maximum protection, high coverage and high on-time immunization levels are equally important. The epidemiology of vaccines has emphasized both individual and herd immunity [5]. When vaccination is delayed, it not only endangers the individual child but also the whole community. Apart from the specific protection acquired from specific vaccines, several studies have demonstrated possible additional beneficial non-specific effects of routine vaccines ([20], [1] [2], [1,30,34,36,39]) thus any delay in vaccinating children means the child will not be getting the extra benefit.

There have been concerted efforts to increase coverage in underserved populations and pockets of unvaccinated children in remote districts by the Equity Reference Group (ERG) which reviews new innovative ideas and approaches to reduce inequities [37]. Studies have documented levels of full- and on-time- vaccination in different settings. A study conducted in urban informal settlements estimated full immunization coverage at 67% and out-of-sequence vaccination at 22% [32]. However, no study has documented the inequality that exists with on-time vaccination in marginalized settings. Thus this study examines the levels and trends of full vaccination and on-time vaccination coverage as well as assessing their associated inequalities in two informal urban settlements of Nairobi.

## Methods

### Study design and setting

The study used data collected from two urban informal settlements (Viwandani and Korogocho) in Nairobi, Kenya, where the African Population and Health Research Center (APHRC) runs the Nairobi Urban Health and Demographic Surveillance System (NUHDSS), with a population of more than 70,000 residents. The two settlements are characterized by poor housing, lack of basic infrastructure, violence, insecurity, high unemployment rates, and poor health indicators [3]. The NUHDSS has been systematically collecting data on vital demographic events, including births, deaths, and migrations occurring among residents of all households in the surveillance area since 2003. Additionally, the NUHDSS has been collecting data on routine childhood immunization for all children under the age of five years. More information regarding the study area and the NUHDSS can be found elsewhere [12]. Three rounds of data collection were conducted each year between 2003 and 2015 before the change to two rounds per year from 2015 onwards. A vaccination tool was administered either during birth registration or when a child in-migrated to the study area. Vaccination information was collected from a vaccination card whenever it was possible and recorded into a data collection questionnaire. When a vaccination card was not produced, the guardians of the child were asked to recall whether the child was given each of the routine vaccines and the missing status of the vaccination card was also recorded.

### Outcome measures

The outcome of interest for this study was a) fully immunized child (FIC) defined as a child aged 12–59 months who have received all basic routine vaccines - one dose of Bacille Calmette-Guérin vaccine, one dose of measles vaccine, three doses of pentavalent vaccine against diphtheria, tetanus, and pertussis (DTP) and three doses of polio vaccine by the age of 12 months b) on-time vaccination, defined as a child aged 12–59 months who received all basic routine vaccines within four weeks of their scheduled time (Table 1). Data from the NUHDSS was used for this study. Children aged 12–59 months of age, had a vaccination card seen and had received all the 8 antigens that were considered for the on-time vaccination analysis. Children below 12 months were excluded from the assessment as some were not not old enough to receive the recommended vaccine or they were still within the age of receiving the vaccine (specifically the MCV vaccine which can be given between the age of nine and twelve months).

**Table 1:** Definition of coverage and on-time.

Outcome	Definition	Indicator
Full immunization overage (FIC)	Received 8 antigens by 12 months of age;	1 if FIC
	1 BCG, 3 OPV, 3 Penta and 1 MCV	0 if not FIC
On-time immunization	a) BCG given within the first 28 days of life	1 if a & b & c & d & e
	&	are true
	b) Penta 1 and OPV1 given between 38 and 70 days (4 days before and 28 days after the recommended age for the vaccine (42 days))	
	&	0 if either a|b|c|d|e
	c) Penta 2 and OPV2 given between 66 and 98 days (4 days before and 28 days after the recommended age for the vaccine (70 days))	is false
	&	
	d) Penta 3 and OPV3 given between 94 and 126 days (4 days before and 28 days after the recommended age for the vaccine (98 days))	
	&	
	e) Penta 3 and OPV3 given between 94 and 126 days (4 days before and 28 days after the recommended age for the vaccine (98 days))	
	&	
	f) The first dose of measles vaccine given between 270 and 298 days (4 days before and 28 days after the recommended age for the vaccine (270 days))	

### Dimensions of inequality

Four dimensions of inequality (difference in coverage among specific sub-populations) were considered; socioeconomic, sex, settlement area, and ethnicity. Principal component analysis technique was used to compute the wealth index using household possessions, materials used for housing construction, and available infrastructures such as types of water access and sanitation facilities. The wealth index was then grouped into five equal subgroups (quintiles) with the first quintile defining the poorest households and the fifth quintile representing the richest households. The grouping was done at the household level, hence the households sampled for this study may not necessarily be divided into five equal groups. We also explored inequality by sex and the two informal settlement areas. Viwandani is located in an industrial area within the city of Nairobi and most of the residents are young working adults working within the area and most have better economic and health indicators as compared with Korogocho which is a more settled area. Differences in vaccination coverage by ethnicity which has been shown in other studies to influence most health indicators were also examined. Coverage trends were assessed annually from 2003 to 2015 (years with available data). Due to inadequate sample size in some years, analysis were repeated by grouping visit years into five groups (2003, 2004-2006, 2007-2009, 2010-2012 and 2013-2015).

### Statistical analysis

Absolute difference (AD) in percentage points and the relative ratio (RR) between the two extreme values of each dimension of inequality measures were used to summarize the levels of inequality. These are simple comparisons of full and on-time immunization coverages recorded for the extreme values e.g. lowest wealth quintile and that recorded for the highest wealth quintile. We then used equiplots to assess and visualize the levels of inequalities. The simple comparison ignores the whole distribution of the inequality dimension, hence we used the more advanced method which takes into consideration the whole distribution of the particular dimension of inequality; the slope index of inequality (SII) – which uses a logistic regression model to express the absolute difference in coverage, in percentage points, between the extremes of the wealth distribution and concentration index (CIX) that is similar in concept to the Gini index for income distribution [18,43]. The concentration index is expressed on a scale from −100 to +100, with full equality indicated by a value of zero. Standard errors for each summary indicator and corresponding *p*-values for the probability that there was no inequality were calculated. The absolute difference and SII indicators give an idea of the effort needed to close the gap. Relative ratio and the concentration index indicators provide the degree of disparity. Population attributable fractions (PAF) for selected inequality factors were calculated to quantify the contribution of the factor to under- and/or untimely vaccination. We used multivariate logistic regression framework to estimate the PAF using Miettinen's formula [29]. The model allows the control of confounders and hence bias which is one of the assumptions of PAF. The higher the PAF, the greater the proportion of the outcome that is attributable to the risk factor.

Multiple failure-time data (recurrence) or multivariate survival data occur when two or more events (failures) occur for the same individual, or when events occur among individuals who are clustered within the same family or household. The failure times are correlated within an individual or cluster, which violates the independence of failure times [21]. Therneau and Hamilton suggested that failure events should be classified according to their natural order of occurrence, should there be one, and within their same reoccurrence types [38] to give robust standard errors. We used multivariate survival analysis techniques to assess the time to full- and on-time vaccination. Time to each of the basic recommended routine childhood vaccine was used to describe the survival data. By definition, vaccines should be given at specific ages and in a particular sequence (ordered), but in practice vaccines can be given in an unorderly manner. The follow up time was from the date the child was born. A child was considered censored if they did not get vaccinated with a particular antigen by the age of one year (for FIC) and if they did not get vaccinated on scheduled time for a particular antigen by the age of one year (for on-time vaccination). 11.5% and 35.6% of the children records were censored for time to FIC and to on-time vaccination analyses respectively. The median follow up time for the time to FIC analysis was 94 days IQR (48, 169) and for time to on-time vaccination was 79 days IQR (46, 118).

We made use of all available data while accounting for the lack of independence of the failure times by including a random-effect term to model unobserved effects shared by all members of the cluster while accounting for within-group dependency. These random effects can be included multiplicatively in the hazard function as shared frailty. A shared frailty Cox model was fitted to account for the heterogeneity due to multiple vaccinations. Frailty is a latent random effect that enters multiplicatively on the hazard function. In a Cox model, the data are organized as *i* = 1 … n groups with j = 1…n_i_ observations in group i. For the j^th^ observation in the i^th^ group, the hazard is

$$h_{\mathrm{ij}}\left(t\right)=h_{0}\left(t\right)\alpha_{i}\exp\left(X_{\mathrm{ij}}\beta \right)$$

where *α*_*i*_ is the group-level frailties which are unobservable positive quantities and are assumed to have mean 1 and variance *θ*, to be estimated from the data. Shared-frailty models are used to model within-group correlation; observations within a group are correlated because they share the same frailty. The estimate of *θ* is used to measure the degree of within-group correlation, and the shared-frailty model reduces to standard Cox when *θ* = 0. For *ϑ*_*i*_ =log*α*_*i*_, the hazard can also be expressed as

$$h_{\mathrm{ij}}\left(t\right)=h_{0}\left(t\right)\exp\left(X_{\mathrm{ij}}\beta +\vartheta_{i}\right)$$

and thus the log frailties, *ϑ*_*i*_, are analogous to random effects in standard linear models. Likelihood ratio test of *H*_0_: *θ* = 0 was used to test correlation within a child. Bivariated models were first fitted to assess the effect of each variable on time to full- and on-time vaccination coverage respectively. All available variables were then added to the multivariate models to assess their effects after controlling other variables. We used partial Cox–Snell residuals to assess overall model fit for each record within a child and Schoenfeld and scaled Schoenfeld residuals to test the proportional-hazards assumption.

## Results

A total of 32,018 children aged between 12 and 59 months who had at least one household visit after the age of 12 months were considered for this analysis. Almost all (98%) children reported having ever had a vaccination card with less than half (46.7%) of the respondents producing a vaccination card during the interview. These children were included in the on-time vaccination analysis. Vaccination card were seen in about half of the children included in the study throughout the period under consideration, with lowest proportion observed in the period 2013–2015 (41.4%) (Table 2). The proportion of children where a vaccination card was seen was lowest (31.8%) in 2009 and the highest proportion (61.8%) in 2008 (Supplementary materials Table 1). The sampled population consisted of a similar number of children from both study sites apart from the latter years where slightly more children from Viwandani than Korogocho were included. The study participants included slightly more children from Kikuyu ethnic group throughout the period of survey as compared to the other ethnic groups. The sample also included considerably more children from the wealthiest quintile. A significantly higher proportion of vaccination cards were seen among children from Korogocho settlement area and from wealthiest households compared to Viwandani and from the poorest households respectively. Significant differences were also observed by ethnic groups over the survey periods (Supplementary materials Table 2). The differences were not different by ethnicity and sex.

**Table 2:** Background characteristics of study participants by period of survey.

Factors	Year of visit				
	2003	2004–2006	2007–2009	2010–2012	2013–2015
Has vaccination card	97.7	98.2	91.4	93.4	95.6
Vaccination card seen	49.0	55.3	48.1	49.0	41.4
Site					
Korogocho	50.7	51.4	50.2	47.6	43.6
Viwandani	49.3	48.6	49.8	52.4	56.4
Sex					
Female	49.9	49.2	50.3	50.2	50.4
Male	50.1	50.8	49.7	49.8	49.6
Ethnicity					
Kikuyu	28.4	27.4	28.4	25.6	20.8
Luhya	16.9	17.1	18.1	20.6	23.2
Luo	21.6	21.4	18.0	16.1	15.3
Kamba	18.8	20.2	20.9	21.4	22.8
Others	14.1	13.7	14.5	16.2	17.7
Househols wealth quintiles					
Quintile 1 (poorest)	26.8	21.2	14.2	13.7	12.0
Quintile 2	11.5	16.4	16.2	16.7	16.7
Quintile 3	13.3	19.3	18.3	19.1	19.0
Quintile 4	18.6	19.3	22.4	22.6	23.5
Quintile 5 (richest)	28.8	23.4	26.2	25.5	24.3
N	6290	6718	7254	7338	4418

### Full immunization coverage (FIC)

**Fig. 1:**
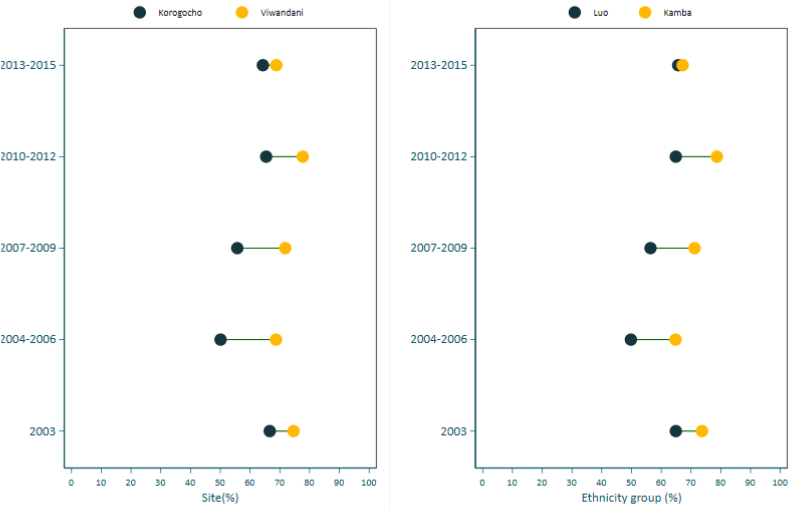
Levels and inequities of full immunization coverage (FIC) among urban poor by site and ethnicity.

Table 3 summarizes overall FIC by survey periods and selected stratifiers (study area, household wealth status, child sex, and ethnicity of the mother. There was a general increase in overall FIC coverage from the early survey period (58.2%, in 2004–2006) to 71.6% during the survey period 2010–2012. FIC coverage was consistently high in the Viwandani study site compared to Korogocho throughout the years. FIC coverage was highest among Kikuyu and Kamba ethnic group compared to other groups. There were no differences in FIC coverage between girls and boys. FIC coverage was consistently high among respondents from the wealthiest households compared to those from the poorest households except for the period 2013–2015. Fig. 1 visualizes the trends in coverage over the years in the study site by ethnicity and site location. No clear pattern in coverage by wealth quintiles and sexes over the years was observed (results not presented). A clear difference in coverage between the two sites was observed with Viwandani consistently having higher coverage rates than Korogocho. The coverage difference was higher during the periods between 2004 and 2012 but narrowed (improved) during the latest survey period (2013–2015). In terms of ethnicity, coverage was consistently higher among the Kamba ethnic group compared to the Luo ethnic group.

**Table 3:** Proportion of fully immunized children by survey period and selected stratifiers.

Stratifiers	Year of visit				
	2003	2004–2006	2007–2009	2010–2012	2013–2015
	%	%	%	%	%
Site					
Korogocho	66.6	50.1	55.7	65.4	64.3
Viwandani	74.6	68.7	71.8	77.7	68.8
Wealth quintiles					
Quintile 1 (poorest)	68.6	55.8	58.8	70.5	67.8
Quintile 2	68.8	57.5	66.1	73.6	67.7
Quintile 3	68.8	62.8	65.3	70.5	68.0
Quintile 4	66.4	56.3	61.9	70.5	63.4
Quintile 5 (richest)	73.9	58.3	65.9	72.0	67.8
Sex					
Female	69.5	57.3	62.9	72.2	67.6
Male	69.9	58.9	65.2	71.0	65.7
Ethnicity					
Kikuyu	75.7	63.9	68.2	73.3	65.2
Luhya	64.9	57.4	59.8	70.0	68.1
Luo	64.9	49.8	56.4	64.9	65.7
Kamba	73.7	64.8	71.2	78.7	67.2
Others	67.6	53.9	60.1	68.3	67.0
Overal	69.7	58.2	64.0	71.6	66.6
N	3081	3718	3491	3596	1828

The above differences are also confirmed by the absolute differences (AD) and relative ratio (RR) (see supplementary materials Table 3), a positive absolute difference was observed between Viwandani and Korogocho sites indicating a higher FIC coverage in Viwandani with highest AD observed in the period 2004–2006 (18.6 pp) and 2007–2009 (16.1 pp) and lowest during the latest survey period 2013–2015. The relative ratio between Viwandani and Korogocho was above one indicating an inequality favoring the Viwandani site. Positive absolute differences by wealth status were observed in all survey periods indicating higher coverage in the advantaged subgroup (quintile 5) except for the latest survey period 2013 to 2015. There was no clear inequality observed by the sex of the child. A positive absolute difference was observed between Kikuyu and Luo ethnic groups indicating a higher FIC coverage among Kikuyus with the highest AD in the period 2004–2006. The relative ratio between Kikuyu and Luo was above one indicating an inequality favoring Kikuyu ethnic group.

Table 4 below summarizes the population attributable fractions (PAF) for non-FIC and untimely immunization by selected stratifiers and survey period (Table 4). The PAF analysis showed that between 2.8% and 8.3% of all cases of non-FIC could be attributed to being residence of Korogocho. Korogocho's PAF decreased from 8.2% (95% CI: 6.7, 9.7) during the period 2004–2006 to 3.1% (95% CI: 0.5, 5.7) in the period 2013–2015 showing improvement in inequality reduction over the years. Being FIC could also be attributed to being from Kikuyu ethnic group (PAF estimates ranging from −6.6% to −2.7% over the period) indicating protective effects interpreted as the proportion increase expected in the number children not fully immunized if the protective exposure was absent from the population. The Luo and others ethnic group accounted for the largest positive PAF of being non-FIC in the study area. The results showed a significant PAF for non-FIC attributed to respondents from poorest households during the periods 2004–2009. The PAF estimates for Viwandani were all negative for the fully immunized coverage analysis indicating protective effect. Supplementary materials Table 4 summarizes the results of the concentration index (CIX) for full immunization coverage by sex, wealth, residence, and ethnicity by survey period. Positive CIX values are observed for the place of residence for all the survey periods indicating FIC coverage was higher among respondents from Viwandani. CIX by ethnicity were all negatives and minimal indicating higher FIC coverage among respondents from the Kikuyu ethnic group. CIX for wealth status was all positive apart from the last survey period indicating higher FIC coverage among respondents from the wealthiest households. The SII for the four stratifiers under consideration (results not shown) were all estimated at zero for all the years under consideration implying no significant inequality observed.

**Table 4:** Population attributable fraction for selected factors associated with non-full immunization and untimely immunization, by survey periods.

	Factors	Surveys periods				
		2003 PAF% (95% CI)	2004–2006 PAF% (95% CI)	2007–2009 PAF% (95% CI)	2010–2012 PAF% (95% CI)	2013–2015 PAF% (95% CI)
Full immunization coverage	Place of residence					
	Koch	2.8 (1.4; 4.1)	8.2 (6.7; 9.7)	8.3 (6.4; 10.1)	5.5 (3.9; 7.2)	3.1 (0.5; 5.7)
	Viwa	−4.4 (−6.6; −2.3)	−10.8 (−12.7; −8.9)	−7.7 (−9.3; −6.0)	−5.7 (−7.3; −4.0)	−2.8 (−5.0; −0.5)
	Child's gender					
	Female	0.1 (−1.5; 1.7)	0.8 (−0.8; 2.4)	1.2 (−0.4; 2.8)	−0.8 (−2.3; 0.6)	−1.0 (−3.1; 1.2)
	Male	−0.1 (−1.8; 1.6)	−0.8 (−2.3; 0.7)	−1.2 (−2.8; 0.4)	0.9 (−0.6; 2.4)	1.0 (−1.2; 3.2)
	Mother's Ethnicity					
	Kikuyu	−6.0 (−8.4; −3.5)	−6.6 (−9.1; −4.1)	−5.7 (−8.2; −3.2)	−2.7 (−5.2; −0.1)	1.4 (−3.3; 6.1)
	Luhya	4.2 (0.6; 7.8)	−0.3 (−3.7; 3.1)	3.1 (−0.3; 6.4)	0.2 (−2.5; 3.0)	−2.0 (−5.8; 1.7)
	Luo	3.0 (0.0; 6.0)	3.9 (1.0; 6.8)	3.6 (0.1; 7.1)	3.5 (0.1; 6.9)	−0.7 (−5.6; 4.3)
	Kamba	−1.2 (−5.4; 2.9)	1.0 (−2.9; 4.9)	−2.0 (−5.4; 1.4)	−3.4 (−6.5; −0.2)	1.0 (−3.4; 5.3)
	Other	2.6 (−1.3; 6.5)	5.5 (1.8; 9.2)	5.8 (1.9; 9.7)	4.7 (0.9; 8.5)	0.9 (−4.3; 6.2)
	Household wealth status					
	Quintile 1 (poorest)	1.7 (−1.1; 4.5)	3.7 (0.5; 6.9)	5.7 (1.4; 9.9)	1.9 (−2.3; 6.0)	−1.0 (−7.0; 5.1)
	Quintile 2	0.6 (−3.9; 5.1)	−0.9 (−4.8; 2.9)	−1.6 (−5.3; 2.1)	−2.1 (−5.3; 1.1)	−1.3 (−6.3; 3.6)
	Quintile 3	0.9 (−3.3; 5.0)	−5.4 (−8.7; −2.1)	−2.2 (−5.4; 1.0)	−0.2 (−3.1; 2.8)	−1.3 (−5.6; 3.0)
	Quintile 4	3.0 (−0.4; 6.4)	1.2 (−1.9; 4.3)	1.7 (−1.3; 4.7)	0.9 (−1.8; 3.6)	3.6 (−0.2; 7.3)
	Quintile 5 (richest)	−4.0 (−6.4; −1.6)	0.5 (−2.0; 3.0)	−1.3 (−3.7; 1.1)	−0.1 (−2.6; 2.3)	−1.3 (−5.0; 2.4)
Timely immunization coverage	Place of residence					
	Koch	0.1 (−1.6; 1.8)	6.5 (4.4; 8.5)	7.4 (4.9; 9.8)	5.1 (2.7; 7.4)	3.7 (0.3; 7.1)
	Viwa	−0.1 (−2.5; 2.3)	−6.2 (−8.3; −4.2)	−5.2 (−7.0; −3.4)	−4.2 (−6.1; −2.2)	−3.1 (−5.8; −0.3)
	Child's gender					
	Female	0.3 (−1.5; 2.2)	−0.9 (−2.8; 1.0)	−0.3 (−2.3; 1.7)	−0.2 (−2.1; 1.7)	−3.3 (−6.0; −0.7)
	Male	−0.4 (−2.3; 1.6)	0.8 (−1.0; 2.6)	0.3 (−1.6; 2.3)	0.2 (−1.8; 2.1)	3.6 (0.7; 6.5)
	Mother's Ethnicity					
	Kikuyu	−5.2 (−8.1; −2.3)	−7.3 (−10.3; −4.3)	−10.7 (−13.8; −7.5)	−6.8 (−10.2; −3.5)	−6.7 (−12.7; −0.6)
	Luhya	0.3 (−3.9; 4.6)	7.4 (3.5; 11.2)	10.2 (6.0; 14.4)	9.4 (5.8; 13.0)	11.4 (6.9; 16.0)
	Luo	4.5 (1.1; 8.0)	2.4 (−1.6; 6.4)	6.8 (1.9; 11.6)	3.1 (−1.7; 7.8)	−0.3 (−6.9; 6.4)
	Kamba	2.8 (−1.7; 7.2)	2.0 (−1.9; 5.8)	−0.6 (−4.3; 3.2)	−4.0 (−7.8; −0.2)	−7.1 (−12.5; −1.8)
	Other	0.9 (−3.6; 5.3)	0.8 (−3.7; 5.4)	4.6 (−0.2; 9.3)	1.6 (−3.2; 6.4)	0.5 (−6.0; 7.0)
	Household wealth status					
	Quintile 1 (poorest)	1.7 (−1.5; 4.8)	1.5 (−2.2; 5.3)	3.2 (−2.3; 8.7)	4.2 (−1.0; 9.4)	6.3 (−1.3; 13.8)
	Quintile 2	1.4 (−3.9; 6.6)	2.3 (−2.4; 7.0)	2.1 (−2.4; 6.6)	3.1 (−1.0; 7.2)	6.2 (0.1; 12.3)
	Quintile 3	4.9 (0.3; 9.5)	2.8 (−1.0; 6.6)	3.8 (−0.2; 7.8)	0.8 (−3.1; 4.8)	1.8 (−3.7; 7.2)
	Quintile 4	−2.8 (−6.9; 1.4)	0.3 (−3.4; 4.1)	2.2 (−1.7; 6.0)	−4.6 (−8.1; −1.1)	−5.7 (−10.5; −0.8)
	Quintile 5 (richest)	−2.2 (−5.0; 0.6)	−4.2 (−7.3; −1.2)	−5.9 (−8.9; −3.0)	−0.5 (−3.6; 2.6)	−2.7 (−7.3; 2.0)
	PAF - Population attributable fraction					

### On-time immunization coverage

Overall on-time vaccination was generally low (below 50%) throughout the periods of surveys. The on-time coverage increased from a low of 26.9% in 2003 to a higher of 43.4% in the 2010–2012 survey period. On-time vaccination coverage was higher in Viwandani compared to Korogocho over the years under study. On-time vaccination coverage was higher among respondents from the richest wealth quintile compared to the poorest quintile. Timely immunization were higher among respondents from the Kikuyu and Kamba ethnic group as compared to the rest (Table 5). Positive absolute difference (AD) was observed between the richest and poorest quintiles throughout the survey periods (Supplementay Table 4). The difference in on-time vaccination coverage between Viwandani and Korogocho was also positive throughout the surveys favoring the Viwandani study site apart from the 2003. On-time vaccination coverage was also higher among children from Kikuyu ethnic group compared to other ethnic groups. (Table 5).

**Table 5:** Proportion of fully immunized children aged 12–59 who received their vaccination ontime, by period of survey visit and stratifiers.

Stratifiers	Year of visits (%)				
	2003	2004–2006	2007–2009	2010–2012	2013–2015
Site					
Korogocho	27.0	21.7	30.5	37.2	37.0
Viwandani	26.7	34.1	42.7	48.4	46.4
Wealth quintiles					
Quintile 1 (poorest)	25.1	27.4	34.4	39.9	35.8
Quintile 2	25.2	24.5	34.9	40.1	35.2
Quintile 3	21.9	24.5	32.9	40.8	39.3
Quintile 4	29.9	26.9	35.0	48.0	48.1
Quintile 5 (richest)	29.3	33.3	44.8	45.0	44.9
Sex					
Female	26.4	28.8	38.2	43.2	45.6
Male	27.4	27.2	37.0	43.4	38.3
Ethnicity					
Kikuyu	32.3	34.5	47.2	49.9	49.3
Luhya	25.9	20.1	26.3	32.8	29.5
Luo	22.4	22.7	28.3	37.0	40.8
Kamba	24.1	29.5	41.0	50.3	50.5
Others	26.3	29.5	35.5	42.8	42.9
Overall	26.9	28.0	37.6	43.4	42.1
N	2148	2161	2233	2572	1218

**Fig. 2:**
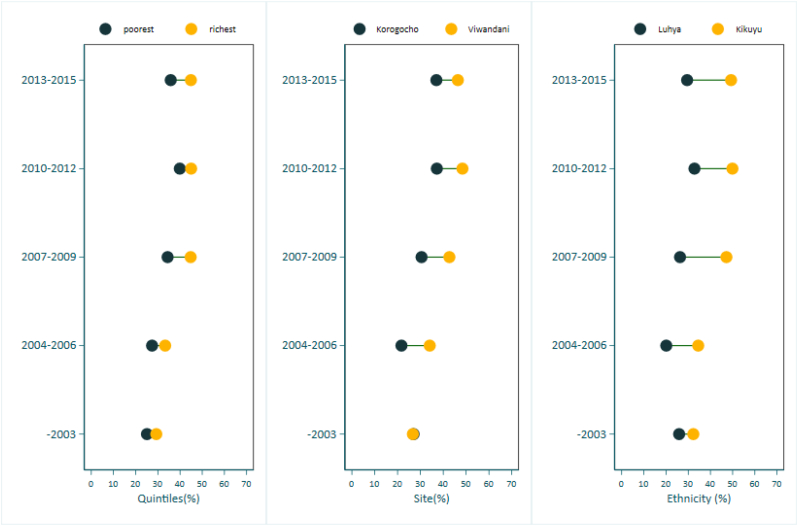
Levels and inequities of timely immunization among urban poor by wealth, site, sex and ethnicity.

PAF estimates for untimely immunization coverage (Table 4) shows a significant proportion of untimely immunization coverage could be attributed to being resident of Korogocho. Korogocho's PAF decreased from a high of 7.4% (95% CI: 4.9, 9.8) in 2007–2009 to 3.7% (95% CI: 0.3, 7.1) in 2013–2015. The PAF estimates for Viwandani were all negative for the on-time immunization coverage analysis indicating protective effect. Timely immunization could also be attributed to being from Kikuyu ethnic group (PAF ranging from −10.7 to −5.2%) - the proportion increase expected in the number children not immunized on-time if the protective exposure was absent from the population. The Luhya and Luo ethnic groups accounted for the largest positive PAF of being untimely immunized in the study area. The results showed a significant PAF for timely immunization attributed to respondents from richest households during the periods 2004–2009. Supplementary materials Table 4 summarizes the results of the concentration index (CIX) for full immunization coverage by sex, wealth, residence, and ethnicity by survey period. Positive CIX estimates were observed by wealth status while negative patterns were observed for ethnicity. The trends in on-time vaccination coverage by the different stratifiers over the years are presented in Fig. 2. No clear pattern in on-time coverage was observed by wealth quintiles or sexes over the years observed. There was a visible on-time vaccination coverage difference between the two study sites with Viwandani consistently having higher coverage rates compared to Korogocho. The coverage gap improved over the years. In terms of ethnicity, on-time coverage was consistently higher among Kikuyu and Kamba ethnic groups compared to the rest and it seems to be narrowing over the years.

### Cox model estimates

The estimated thetas (θ) for the adjusted frailty models for time to -full immunization and -on-time vaccination coverages were estimated at 2.48 and 0.71 which are the variances of the random effects and indicate a substantial within-group correlation. The time to vaccination (FIC and on-time) for given children are correlated. There were no significant differences in terms of both full and on-time immunization coverage by sex of the child. The time to full immunization after accounting for intragroup correlation via a shared frailty model, for a given level of frailty the hazard for being FIC was less likely for for 2004–2006 (23%), 2007–2009 (11%) and 2013–2015 (10%) as compared to the baseline year (2003). The difference was not significantly different for the period 2010–2012. The time to full immunization coverage after accounting for intragroup correlation via a shared frailty model, for a given level of frailty the hazard for being FIC was 10% more likely for children from the wealthiest households as compared to those from poorest households. The hazard for being FIC was 16%, 16%, 7% and 19% less likely for Luhya, Luo, Kamba and other ethnic groups as compared to the Kikuyu ethnic group respectively.

The time to on-time immunization coverage after accounting for intragroup correlation via a shared frailty model, for a given level of frailty the hazard for being timely vaccinated was between 10% significantly less likely for the initial survey period (2004–2006) as compared to the baseline year (2003). The hazard of being timely vaccinated was however 8%, 24% and 18% significantly more likely for the subsequent survey periods 2007–2009, 2010–2012 and 2013–2015 respectively as compared to the baseline year (2003). The time to on-time vaccination coverage after accounting for intragroup correlation via a shared frailty model, for a given level of frailty the hazard for being vaccinated on time was 14% more likely for children from the wealthiest quintile as compared to the poorest quintile. The hazard for being vaccinated on time was 24%, 23%, 11% and 23% less likely for Luhya, Luo, Kamba and other ethnic groups as compared to the Kikuyu ethnic group respectively. Table 6 below summarizes the results above.

**Table 6:** Cox frailty model for time to full immunization and to on-time vaccination by 12 months of age.

Factors	Time to FIC by 12 months		Time to on-time immunization coverage	
	HR [95%CI]	*P* value	HR [95%CI]	P value
Sex (Ref:Female)	1.00		1.00	
Male	0.99 [0.94; 1.03]	0.586	0.99 [0.96; 1.02]	0.510
Year of visit (Ref: 2003)	1.00		1.00	
2004–2006	0.77 [0.72; 0.84]	<0.001	0.90 [0.86; 0.95]	<0.001
2007–2009	0.89 [0.83; 0.96]		1.08 [1.03; 1.13]	
2010–2012	1.02 [0.94; 1.10]		1.24 [1.18; 1.30]	
2013–2015	0.90 [0.83; 0.99]		1.18 [1.12; 1.25]	
Study site (Ref: Korogocho)	1.00		1.00	
Viwandani	1.31 [1.23; 1.38]	<0.001	1.30 [1.26; 1.35]	<0.001
Household Wealth status (Ref: Quintile 1 (poorest))	1.00		1.00	
Quintile 2	1.06 [0.97; 1.15]	0.015	1.06 [1.00; 1.12]	<0.001
Quintile 3	1.11 [1.02; 1.20]		1.09 [1.03; 1.14]	
Quintile 4	1.02 [0.94; 1.10]		1.07 [1.02; 1.13]	
Quintile 5 (richest)	1.10 [1.02; 1.19]		1.14 [1.09; 1.20]	
Ethnicity (Ref: Kikuyu)	1.00		1.00	
Luhya	0.84 [0.78; 0.91]	<0.001	0.76 [0.72; 0.79]	<0.001
Luo	0.84 [0.77; 0.90]		0.77 [0.73; 0.81]	
Kamba	0.93 [0.86; 1.01]		0.89 [0.85; 0.94]	
Other	0.81 [0.74; 0.88]		0.77 [0.73; 0.81]	
Theta	2.48 [2.41; 2.54]		0.71 [0.69:0.73]	

## Discussion

This is the first study to provide estimates for the inequality that exists with on-time vaccination coverage in marginalized settings. The study findings show a high existence of inequalities within the most disadvantaged populations highlighting mothers from certain ethnic communities, children in the poorest wealth quintile and children from the Viwandani slum to be disadvantaged in vaccine coverage, and suggest more focused and targeted interventions for these groups.

A general increase in FIC and timely immunization coverage over time in the study area were observed. FIC increased during the early periods and slightly decreased during the last few years of the surveys included in the study. An increasing FIC trend was also observed in other studies around the same period [[22], [23], [24]] [[40], [44], [47]] in other countries. This increase in FIC observed was likely due to the increase in immunization coverage during the early period of the 2000s [[40], [44], [47]] which coincided with the beginning of the decade of vaccines and the ratification of the GVAP [45]. The decrease in vaccine coverage in the mid-2000s coincides with the introduction of the cost-sharing policy introduced by GAVI in which countries were required to increase their funding to immunization programs as their economies grew [15]. Timely immunization coverage was low during the early period of the study but the on-time coverage improved considerably. This is likely due to the campaigns and immunization programme activities in the study area.

Higher coverages (FIC and timely) was observed and attributed to Viwandani compared to Korogocho. This coverage difference has been documented by other studies conducted in the same study area [11,31] and for other health indicators such as infants feeding practices [25] and stunting [14]. The higher coverage in Viwandani can be attributed to the fact that it is located next to an industrial area and thus residents have access to daily livelihood [12] and by extension access to more healthcare services as compared to Korogocho. The coverage gap between the two sites slightly reduced between 2004 and 2012 and this could have been attributed to the concerted efforts by the international communities together with the national and county governments to reach the unreached population as well as the introduction of new vaccines in the last decades. The reduced gap between the two sites during the 2013–2015 survey period can be attributed mainly to the sharp reduction in FIC coverage in the Viwandani site.

Inequality in full and timely coverage was observed by maternal ethnicity. Coverage was significantly higher among children from Kikuyu and Kamba ethnic groups compared with the other ethnic groups. Similar results were found in other studies conducted in Kenya, and this finding is also visible when looking at other health indicators [11,13,31]. Cultural differences, education and income disparities among the different ethnicities have been cited to be the cause of the ethnic group differences observed in vaccine coverage in slum settings [11,13,25,31]. Similar inequalities by ethnicity has been observed in other parts of SSA [7,27,35]. The gap between the ethnic groups reduced sharply during the 2013–2015 survey period, which was mainly due to a sharp reduction in FIC coverage among the Kikuyu and Kamba ethnic group.

The study also established inequality based on the social-economic status of the households, high FIC, and on-time coverage was observed among the wealthiest quintile as compared to the lowest quintile. This has been shown previously with studies in the same area [11,13,31,32] as well as other settings [9,17,19] suggesting the existence of an access problem with vaccination in the area. The frailty model estimates show significant differences in full and on-time coverage by study site, household wealth status, and mother ethnicity after controlling for a significant intragroup variability and other variables in the model.

The study has both strengths and limitations. A major strength of this study is the use of data from longitudinal health and demographic surveillance system which has been collecting data over the years. This platform has enabled estimation of the coverage gaps by factors as well as assessing the trends over the years. The study is based on one of the most disadvantaged and hard-to-reach populations in an urban area which is normally known for low uptake of most health interventions. The main limitation of this study is the exclusion of children who did not present a vaccination card at the time of survey visits specifically because of the timeliness analysis. This introduces potential selection bias in the analysis and may underestimate or overestimate the true coverage rates as it tends to be more frequent among the most underserved groups as observed in this study. The low proportion of vaccination card seen though is comparable to other studies which collect vaccination data; the Kenya Demographic and Health Surveys (KDHS) surveys conducted during the same period, KDHS 2003 [22], KDHS 2008 [23], KDHS 2014 [22] where 47.7%, 41.5% and 61.7% of the respondent's vaccination card were seen during the time of interview visit in Nairobi county respectively. The paper is based on data collected from informal urban settlements and therefore the results may not be generalizable to the whole population in Nairobi.

In conclusion, the study has been able to document levels and trends of full immunization coverage as well as timeliness of immunization in an informal settlement area. The study has shown that coverage has been increasing over the years. The results from this study are important for local immunization programs as they highlight the existence of inequalities in immunization coverage within the most disadvantaged populations by ethnicity, socio-economic status and residence. The results thus support an urgent need to develop new strategies, interventions and programs that will expand access to the hard to reach populations in order to reduce the levels of under vaccination while eliminating the gap between subgroups.

## Ethical consideration

The NUHDSS received ethical clearance from the Kenya Medical Research Institute (KEMRI) in 2002 and it's consequently renewed over the years. This study has used pre-existing data from the NUHDSS which had already received ethical clearance. The analysis was based on an anonymized dataset with no identifiable information on the study participants.

## Declaration of competing interest

The authors have no potential, perceived, or real conflicts of interest relevant to this article to disclose.

## Appendix A Supplementary data

Supplementary material Image 1
