# Supplementary material for: Do inequalities exist in the disadvantaged populations? Levels and trends of full and on-time vaccination coverage in two Nairobi urban informal settlements
Source: Glob Epidemiol. 2020 Nov;2:100044. doi: 10.1016/j.gloepi.2020.100044 (PMC7756173; doi:10.1016/j.gloepi.2020.100044)
Supplement: Supplementary file 1 — Supplementary material [file mmc1.docx]

**Supplementary Materials**

Supplementary Table 1: Background characteristics of study participants by year of survey

|  | Year of visit | | | | | | | | | | | | |
| --- | --- | --- | --- | --- | --- | --- | --- | --- | --- | --- | --- | --- | --- |
| Factors | 2003 | 2004 | 2005 | 2006 | 2007 | 2008 | 2009 | 2010 | 2011 | 2012 | 2013 | 2014 | 2015 |
| Has vaccination card | 97.7 | 97.3 | 98.8 | 99.0 | 99.2 | 97.9 | 79.2 | 83.9 | 97.2 | 98.4 | 92.1 | 98.4 | 98.3 |
| Vaccination card seen | 49.0 | 53.9 | 53.9 | 58.5 | 48.9 | 61.8 | 31.8 | 35.2 | 53.9 | 56.7 | 44.3 | 38.3 | 41.1 |
| Site |  |  |  |  |  |  |  |  |  |  |  |  |  |
| Korogocho | 50.7 | 52.9 | 46.8 | 53.1 | 43.7 | 52.2 | 51.7 | 48.8 | 49.5 | 45.1 | 46.8 | 41.6 | 39.9 |
| Viwandani | 49.3 | 47.1 | 53.2 | 46.9 | 56.3 | 47.8 | 48.3 | 51.2 | 50.5 | 54.9 | 53.2 | 58.4 | 60.1 |
| Sex |  |  |  |  |  |  |  |  |  |  |  |  |  |
| Female | 49.9 | 48.7 | 50.6 | 48.9 | 48.9 | 50.6 | 50.9 | 50.0 | 48.5 | 52.1 | 49.3 | 50.6 | 52.5 |
| Male | 50.1 | 51.3 | 49.4 | 51.2 | 51.1 | 49.4 | 49.1 | 50.0 | 51.5 | 48.0 | 50.7 | 49.4 | 47.5 |
| Ethnicity |  |  |  |  |  |  |  |  |  |  |  |  |  |
| Kikuyu | 28.4 | 28.3 | 28.0 | 25.7 | 26.9 | 29.5 | 28.0 | 27.1 | 25.0 | 24.7 | 22.9 | 19.3 | 18.9 |
| Luhya | 16.9 | 16.6 | 18.3 | 16.8 | 21.2 | 17.8 | 16.7 | 20.2 | 20.3 | 21.1 | 21.5 | 24.9 | 23.9 |
| Luo | 21.6 | 22.8 | 20.3 | 20.7 | 18.1 | 17.2 | 18.8 | 17.4 | 16.2 | 15.1 | 14.5 | 16.3 | 15.2 |
| Kamba | 18.8 | 18.3 | 21.2 | 21.8 | 21.8 | 19.1 | 22.3 | 20.5 | 21.8 | 21.8 | 21.7 | 22.7 | 25.9 |
| Others | 14.1 | 14.0 | 12.1 | 14.7 | 12.0 | 16.3 | 13.8 | 14.6 | 16.5 | 17.3 | 19.2 | 16.7 | 16.0 |
| Househols wealth quintiles | |  |  |  |  |  |  |  |  |  |  |  |  |
| Quintile 1 (poorest) | 26.8 | 26.7 | 17.9 | 16.7 | 18.3 | 11.7 | 14.6 | 13.6 | 13.4 | 14.3 | 11.7 | 11.9 | 13.2 |
| Quintile 2 | 11.5 | 16.8 | 16.0 | 16.3 | 17.3 | 15.6 | 16.2 | 14.4 | 19.8 | 16.1 | 15.4 | 17.7 | 18.3 |
| Quintile 3 | 13.3 | 17.4 | 22.6 | 19.0 | 19.8 | 17.4 | 18.5 | 20.5 | 17.3 | 19.7 | 17.4 | 19.3 | 22.4 |
| Quintile 4 | 18.6 | 16.8 | 19.9 | 22.1 | 22.0 | 22.5 | 22.5 | 22.3 | 22.0 | 23.1 | 24.2 | 23.3 | 22.9 |
| Quintile 5 (richest) | 28.8 | 21.6 | 23.2 | 25.9 | 22.3 | 30.8 | 23.3 | 26.4 | 25.9 | 24.1 | 25.1 | 24.1 | 21.7 |
| N | 6290 | 2825 | 1758 | 2135 | 1583 | 3047 | 2624 | 2315 | 2355 | 2664 | 1942 | 1730 | 750 |

Supplementary Table 2: Proportion of children aged 12-23 whose vaccination card was seen at the time of interview, by survey period and stratifiers

|  | Year of visits | | | | | | | | | | | |
| --- | --- | --- | --- | --- | --- | --- | --- | --- | --- | --- | --- | --- |
| Stratifiers | 2003 | | 2004-2006 | | 2007-2009 | | 2010-2012 | | 2013-2015 | | 2003-2015 | |
|  | % | P value* | % | P value | % | P value | % | P value | % | P value | % | P value |
| Site |  |  |  |  |  |  |  |  |  |  |  |  |
| Korogocho | 59.3 | <0.001 | 61.3 | <0.001 | 50.5 | 0.018 | 54.9 | <0.001 | 47.0 | <0.001 | 55.4 | <0.001 |
| Viwandani | 38.4 |  | 49.0 |  | 53.4 |  | 49.2 |  | 39.7 |  | 46.5 |  |
| Wealth quintiles |  |  |  |  |  |  |  |  |  |  |  |  |
| Quintile 1 (poorest) | 48.0 | 0.465 | 51.5 | <0.001 | 44.3 | <0.001 | 44.0 | <0.001 | 39.1 | 0.082 | 46.7 | <0.001 |
| Quintile 2 | 47.9 |  | 47.2 |  | 48.8 |  | 51.1 |  | 40.5 |  | 47.6 |  |
| Quintile 3 | 48.2 |  | 51.1 |  | 52.0 |  | 51.6 |  | 43.8 |  | 49.9 |  |
| Quintile 4 | 49.4 |  | 58.5 |  | 50.3 |  | 53.2 |  | 45.5 |  | 51.7 |  |
| Quintile 5 (richest) | 50.8 |  | 65.9 |  | 61.3 |  | 56.2 |  | 44.0 |  | 56.4 |  |
| Sex |  |  |  |  |  |  |  |  |  |  |  |  |
| Female | 50.6 | 0.011 | 54.8 | 0.410 | 52.4 | 0.477 | 52.1 | 0.674 | 43.7 | 0.249 | 51.3 | 0.096 |
| Male | 47.4 |  | 55.8 |  | 51.6 |  | 51.6 |  | 42.0 |  | 50.4 |  |
| Ethnicity |  |  |  |  |  |  |  |  |  |  |  |  |
| Kikuyu | 48.7 | <0.001 | 55.8 | <0.001 | 51.9 | 0.432 | 50.5 | <0.001 | 38.0 | <0.001 | 50.3 | <0.001 |
| Luhya | 51.5 |  | 56.7 |  | 53.6 |  | 56.3 |  | 45.9 |  | 53.2 |  |
| Luo | 53.1 |  | 59.7 |  | 50.0 |  | 53.7 |  | 47.7 |  | 53.6 |  |
| Kamba | 39.5 |  | 46.8 |  | 51.7 |  | 52.0 |  | 45.1 |  | 47.5 |  |
| Others | 53.1 |  | 58.9 |  | 53.2 |  | 46.6 |  | 37.5 |  | 50.2 |  |
| * From Chi square test of independence | | | |  |  |  |  |  |  |  |  |  |

Supplementary Table 3: Absolute difference and the relative ratio between the highest and the lowest categories (extremes) for the site, wealth status, sex, and ethnic grouping

|  | Full immunization coverage | | | | | | | | | | |  | On-time immunization coverage | | | | | | | | | | |
| --- | --- | --- | --- | --- | --- | --- | --- | --- | --- | --- | --- | --- | --- | --- | --- | --- | --- | --- | --- | --- | --- | --- | --- |
|  | Site | |  | Wealth status | |  | Sex | |  | Ethnicity | |  | Site | |  | Wealth status | |  | Sex | |  | Ethnicity | |
| Year | AD | RR |  | AD | RR |  | AD | RR |  | AD | RR |  | AD | RR |  | AD | RR |  | AD | RR |  | AD | RR |
| 2003 | 8.00 | 1.12 |  | 5.30 | 1.08 |  | 0.40 | 1.01 |  | 10.80 | 1.17 |  | -0.3 | 1.0 |  | 4.2 | 1.2 |  | 1.0 | 1.0 |  | 6.4 | 1.25 |
| 2004-2006 | 18.60 | 1.37 |  | 2.50 | 1.04 |  | 1.60 | 1.03 |  | 14.10 | 1.28 |  | 12.4 | 1.6 |  | 5.9 | 1.2 |  | -1.6 | 0.9 |  | 14.4 | 1.72 |
| 2007-2009 | 16.10 | 1.29 |  | 7.10 | 1.12 |  | 2.30 | 1.04 |  | 11.80 | 1.21 |  | 12.2 | 1.4 |  | 10.4 | 1.3 |  | -1.2 | 1.0 |  | 20.9 | 1.79 |
| 2010-2012 | 12.30 | 1.19 |  | 1.50 | 1.02 |  | -1.20 | 0.98 |  | 8.40 | 1.13 |  | 11.2 | 1.3 |  | 5.1 | 1.1 |  | 0.2 | 1.0 |  | 17.1 | 1.52 |
| 2013-2015 | 4.50 | 1.07 |  | 0.00 | 1.00 |  | -1.90 | 0.97 |  | -0.50 | 0.99 |  | 9.4 | 1.3 |  | 9.1 | 1.3 |  | -7.3 | 0.8 |  | 19.8 | 1.67 |
|  | **AD=Absolute difference** | | | | | **RR=Relative ratio** | | | |  |  |  |  |  | **AD=Absolute difference** | | | | | **RR=Relative ratio** | | | |
|  | Viwa - Koch | |  |  |  | Viwa/Koch | | |  |  |  |  |  |  | Viwa - Koch | | |  |  | Viwa/Koch | | |  |
|  | Q5-Q1 | |  |  |  | Q5/Q1 | |  |  |  |  |  |  |  | Q5-Q1 | |  |  |  | Q5/Q1 | |  |  |
|  | Male-Female | |  |  |  | Male/Female | | |  |  |  |  |  |  | Male-Female | | |  |  | Male/Female | | |  |
|  | Kikuyu-Luo | |  |  |  | Kikuyu/Luo | | |  |  |  |  |  |  | Kikuyu-Luhya | | |  |  | Kikuyu/Luhya | | |  |

Supplementary Table 4: Slope index of inequality and concentration index for full immunization coverage and on-time vaccination by year of interview and slected stratifiers

|  | Full immunization coverage | | | | | | | |  | On-time immunization coverage | | | | | | | |
| --- | --- | --- | --- | --- | --- | --- | --- | --- | --- | --- | --- | --- | --- | --- | --- | --- | --- |
|  | Sex | | Site | | Wealth status | | Ethnicity | |  | Sex | | Site | | Wealth status | | Ethnicity | |
| Year | CIX | se | CIX | se | CIX | se | CIX | se |  | CIX | se | CIX | se | CIX | se | CIX | se |
| 2003 | -0.001 | 0.000 | 0.028 | 0.001 | 0.009 | 0.011 | -0.016 | 0.015 |  | -0.009 | 0.0001 | -0.003 | 0.000 | 0.040 | 0.014 | 0.003 | 0.034 |
| 2004-2006 | -0.007 | 0.000 | 0.078 | 0.009 | 0.005 | 0.009 | -0.022 | 0.030 |  | 0.014 | 0.0003 | 0.111 | 0.017 | 0.042 | 0.031 | -0.030 | 0.061 |
| 2007-2009 | -0.009 | 0.000 | 0.063 | 0.006 | 0.013 | 0.013 | -0.024 | 0.023 |  | 0.008 | 0.000 | 0.083 | 0.010 | 0.046 | 0.029 | -0.035 | 0.068 |
| 2010-2012 | 0.004 | 0.000 | 0.043 | 0.003 | 0.000 | 0.004 | -0.017 | 0.018 |  | -0.001 | 0.000 | 0.065 | 0.006 | 0.034 | 0.006 | -0.029 | 0.052 |
| 2013-2015 | 0.007 | 0.000 | 0.017 | 0.000 | -0.005 | 0.006 | -0.002 | 0.003 |  | 0.044 | 0.0027 | 0.056 | 0.004 | 0.061 | 0.007 | -0.054 | 0.053 |
